# A Method to Determine the Minimum Chip Thickness during Longitudinal Turning

**DOI:** 10.3390/mi11121029

**Published:** 2020-11-24

**Authors:** Michal Skrzyniarz

**Affiliations:** Department of Manufacturing Engineering and Metrology, Kielce University of Technology, 25-314 Kielce, Poland; mskrzyniarz@tu.kielce.pl; Tel.: +48-4134-24-417

**Keywords:** turning, minimum chip thickness, micromachining

## Abstract

Micromachining, which is used for various industrial purposes, requires the depth of cut and feed to be expressed in micrometers. Appropriate stock allowance and cutting conditions need to be selected to ensure that excess material is removed in the form of chips. To calculate the allowance, it is essential to take into account the tool nose radius, as this cutting parameter affects the minimum chip thickness. Theoretical and numerical studies on the topic predominate over experimental ones. This article describes a method and a test setup for determining the minimum chip thickness during turning. The workpiece was ground before turning to prevent radial runout and easily identify the transition zone. Contact and non-contact profilometers were used to measure surface profiles. The main aim of this study was to determine the tool–workpiece interaction stages and the cutting conditions under which material was removed as chips. Additionally, it was necessary to analyze how the feed, cutting speed, and edge radius influenced the minimum chip thickness. This parameter was found to be dependent on the depth of cut and feed. Elastic and plastic deformation and ploughing were observed when the feed rate was lower than the cutting edge radius.

## 1. Introduction

The manufacture of precision machine parts for the electrical, electronic, computer, biotechnological, and machine tool industries requires an understanding of the physical phenomena occurring at the microscale [1,2], especially when very low-depth cuts are involved [3,4]. One of the key factors affecting the surface quality in micromachining is the minimum chip thickness [5,6]. This parameter defines the minimum depth of cut at which, under specific cutting conditions, material is removed in the form of chip [7,8].

There are three stages of interaction between the tool and the workpiece [9]: (I) elastic and plastic deformation of the workpiece at *a_p_ < h_min_*; (II) elastic and plastic deformation and ploughing at *a_p_ = h_min_*; (III) shearing (chip formation) at *a_p_ > h_min_*.

Researchers dealing with the minimum chip thickness approach the problem both theoretically and experimentally [10,11]. The depth of cut and other cutting parameters are used to optimize the process. 

Currently, there are many mathematical models used to predict *h_min_*. The most important formulae are summarized in Table 1 [12,13,14,15,16,17,18]. 

The calculation of the minimum chip thickness according to Formula (1) requires the stagnation point (*A*) to be determined, which is defined by the stagnant angle (*θ*) in relation to the edge radius (*r_n_*). The stagnation point is used to determine the zones of interaction between the tool and the workpiece. When the thickness of the material to be removed is below the stagnation point, the cutting process involves ploughing with elastic and plastic deformation of the workpiece surface; however, when the thickness of the material to be removed is above the stagnation point, chip removal occurs [12]. 

In [13], Kawalec proposes a formula (Formula (2)) in which the minimum chip thickness (*h_min_*) depends on the edge radius (*r_n_*) and the coefficient of friction between the tool and the workpiece (*k*), with the latter ranging from 0.1 to 1. Other studies confirm that the coefficient of friction affects the surface roughness [19].

In [14], Grzesik applies the molecular–mechanical theory of friction to formulate a model to predict the minimum chip thickness (3). The strain hardening curve is used to calculate the equivalent strain and shear strength of the material in the adhesive junction. In [20], Grzesik analyzes the phenomena responsible for both partial material separation and chip formation. 

Formula (4) proposed by Yuan et al. assumes that *h_min_* is dependent on the edge radius (*r_n_*), the coefficient of friction between the tool and the workpiece (*µ*), and the force ratio *F_y_/F_x_* [15]. 

In [16,17], the model for *h_min_* (5) assumes that the stagnation point (*A*), based on the stagnant angle (*θ*), is affected by the edge radius (*r_n_*). When the stagnant angle is 45°, the minimum chip thickness ranges between 0.2 and 0.35 *r_n_*. 

The major assumption of model (6) is to maintain constant temperature conditions irrespective of the cutting conditions and to measure the cutting forces that affect the directional coefficients *A_f_* and *A_c_* [18].

The approach proposed by L’vov suggests that *h_min_* = 0.29 *r_n_* at *θ* = 37.6° [21]. In [22], however, *h_min_* is reported to be 0.21 *r_n_*. Yuan et al. show that for aluminum alloys, the parameter *h_min_* ranges from 0.25 to 0.32 *r_n_* [15]. A similar relationship is described in [23], where the minimum chip thickness is 0.25–0.3 *r_n_*.

Although most studies on the minimum chip thickness deal with mathematical models, some researchers approach the problem practically [24]. In [25], the method involves preparing a conical sample with a taper angle of 5°, cutting it along the cone height, and placing the two halves joined end-to-end between the centers. The sample is first ground and then turned. After the cutting operations, the halves are separated and the tool–workpiece interaction zones are analyzed using a workshop microscope.

Other methods used to determine the minimum chip thickness require the application of acoustic emission signals to provide information on the phenomena taking place in the cutting zone, especially material separation [26,27].

The literature on the subject does not mention any practical methods for determining the minimum chip thickness during turning, nor does it analyze the tool–workpiece interaction in relation to the minimum chip thickness based on surface profiles. The aim of this study was to develop an experimental method to determine *h_min_* during longitudinal turning.

## 2. New Method and Test Setup for Determining the Minimum Chip Thickness during Turning

**Figure 1:**
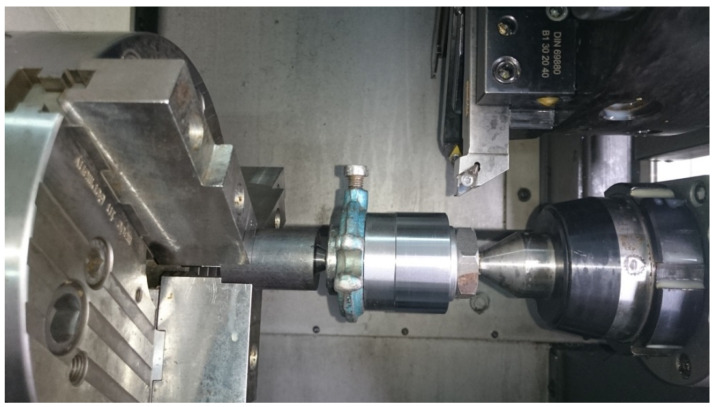
Workpiece holding system used to determine the minimum chip thickness during turning.

The experimental method for determining *h_min_* presented here was developed to overcome the limitations of the approach presented in [25], mainly because of the time- and labor-consuming sample preparation process and the discontinuous cutting, which was interrupted at the point of contact of the two sample halves. The new method uses a conical sample with known convergence (*C* = 0.008). The workpiece is mounted on a mandrel and held in place with a lathe dog at one end and a nut at the other, so there is no need to change the workpiece holding devices. The workpiece system is supported between centers. Grinding is used to produce a tapered surface; it also helps in analyzing the tool–workpiece interactions and to prevent radial run-out during turning so that the material is removed at a constant cut depth around the circumference. The torque is transmitted to the workpiece through the lathe dog. The workpiece holding system used to determine the minimum chip thickness is shown in Figure 1. The turning was performed on a DMG CTX Alpha 500 CNC turning and milling machine.

The method proposed here does not require the cutting process to be interrupted; turning is performed as a continuous process. The sample preparation process is quick and simple. The use of grinding allows us to easily indicate the initial stage of interaction between the tool and the workpiece during turning. The method, however, requires that a concentric workpiece be produced; this means turning needs to be performed at the same cut depth around the circumference.

After turning, the workpiece is analyzed using a contact skidless profilometer (TOPO 01). The primary profile is used as the basis for determining the stages of the tool–workpiece interaction. The sections with regular surface profiles indicate that turning with chip formation has taken place. Profile irregularity suggests that turning with no chip formation has taken place (ploughing). The results obtained in the experiment were accurate and repeatable because the profilometer used is a high-resolution profilometer. The experimental findings were confirmed by 3D surface measurements with a Talysurf CCI optical profilometer.

## 3. Experiment and Results

The experiments were carried out using samples made of X37CrMoV5-1 steel. The material hardness before grinding was 34.9 ± 5 HRC. A Sandvik Coromant CoroTurn 107 SDJCL 2020K 11 turning tool holder was used for longitudinal turning. The inserts tested were 55° diamond-shaped positive turning inserts with a 7° clearance angle and a 93° entering angle. Although manufacturers of cutting tools do not provide information on the edge radius in their product catalogs, this parameter needs to be taken into consideration in micromachining. Three types of standard turning inserts were used in the tests: DCMT 11 T3 08–PF 4325, DCMT 11 T3 08–MF 1105, and DCGT 11 T3 08–UM 1125. The edge radius of each insert was measured before cutting.

**Figure 2:**
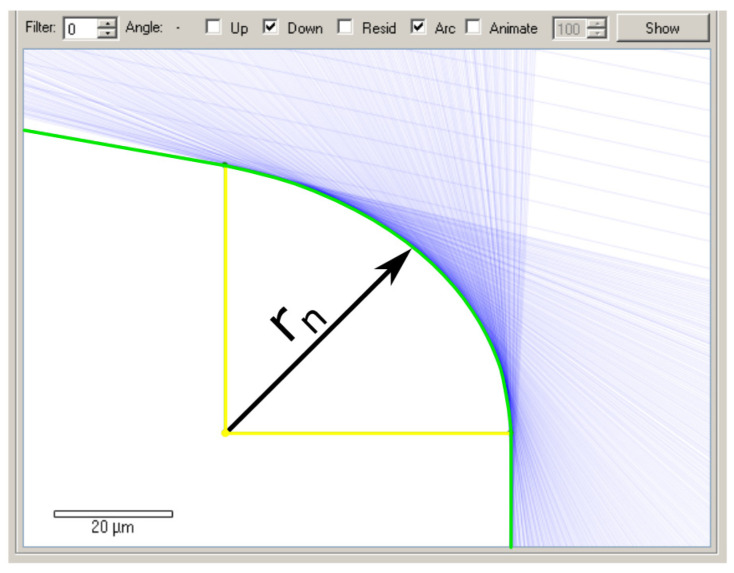
Measurement of the edge radius (*r_n_*) of the DCMT 11 T3 08–PF 4325 insert.

The edge radii were measured using a special setup employed by Sandvik. The measurement involved moving the measuring tip around the cutting edge and recording the data for each position. The edge radius was calculated using the device software. An example of an edge radius measurement report is shown in Figure 2.

Ten inserts were measured for each type of insert to determine their edge radii. For the DCMT 11 T3 08–PF 4325 type, the edge radius values ranged between 44.4 and 55.9 µm; for the DCMT 11 T3 08–MF 1105 type, its values ranged from 29.6 to 38.9 µm; for the DCGT 11 T3 08–UM 1125 type, the values ranged from 5.3 to 11.5 µm.

**Figure 3:**
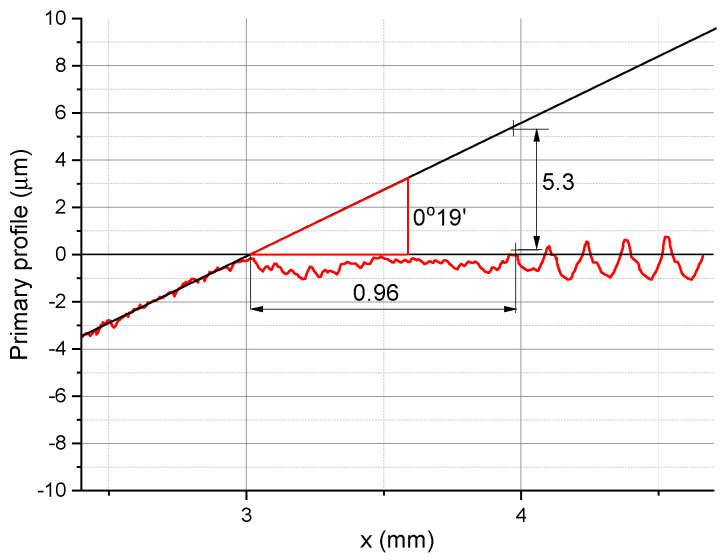
Determining *h_min_* from a surface profile obtained with a contact profilometer (*v_c_* = 360 m/min, *f* = 0.03 mm/rev, and *r_n_* = 49.9 μm).

After turning, the workpiece surface was analyzed with a contact skidless profilometer (TOPO 01). Primary profiles obtained for specific cutting conditions were the basis for determining the minimum chip thickness. Figure 3 shows how the parameter was determined for longitudinal turning.

As can be seen from Figure 3, there are three characteristic zones: the grinding zone, transition zone, and turning zone. In zone 1, there is no interaction between the turning tool and the workpiece. In zone 2, the material undergoes elastic and plastic deformation; partial removal of material also occurs. The irregularity of the profile in this zone indicates an unstable cutting process. The zone is approximately 0.96 mm in length. In order to determine the minimum chip thickness for which chip removal occurred, the ground surface profile was extended as a straight line to find the point indicating the end of zone 2. In the case considered here, i.e., for *v_c_* = 360 m/min, *f* = 0.03 mm/rev, and *r_n_* = 49.9 μm, the minimum chip thickness was 5.3 µm. The regular profile in the third zone represents turning with chip formation.

**Figure 4:**
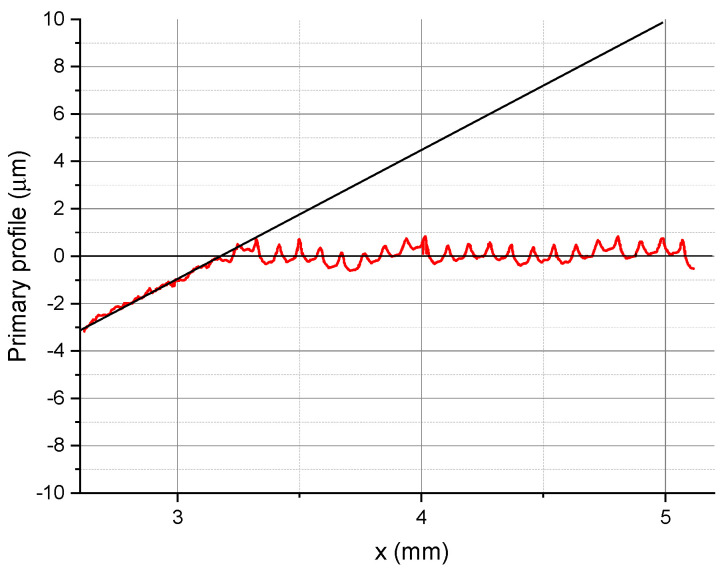
Surface profiles obtained with a contact profilometer for a workpiece machined at *v_c_* = 360 m/min, *f* = 0.09 mm/rev, and *r_n_* = 49.9 μm.

Figure 4 shows the surface profiles obtained at *v_c_* = 360 m/min, *f* = 0.03 mm/rev, and *r_n_* = 49.9 μm. 

From the profile in Figure 4, it is clear that the material did not undergo elastic and plastic deformation, nor was it separated partially (ploughing). Partial removal of material was observed at *f* = 0.03 mm/rev. At feed rates higher than 0.06 mm/rev there was no transition zone between grinding and turning. No unstable cutting was reported. 

When the cutting was performed using a DMCT 11 T3 08–PF 4325 insert with an edge radius of 49.9 µm, elastic and plastic deformation occurred. With feed rates lower than the edge radius *r_n_*, partial removal of material (ploughing) was observed. Feed rates greater than the edge radius ensured material removal in the form of chips. To verify the validity of the method, the experiment was carried out with feed rates ranging from 0.01 mm/rev to 0.06 mm/rev. Table 2 shows the cutting parameters used to determine the minimum chip thickness. The data provided in Table 2 indicate that the unstable cutting process responsible for elastic and plastic deformation and partial material removal (ploughing) was observed with feed rates smaller than the edge radius.

**Figure 5:**
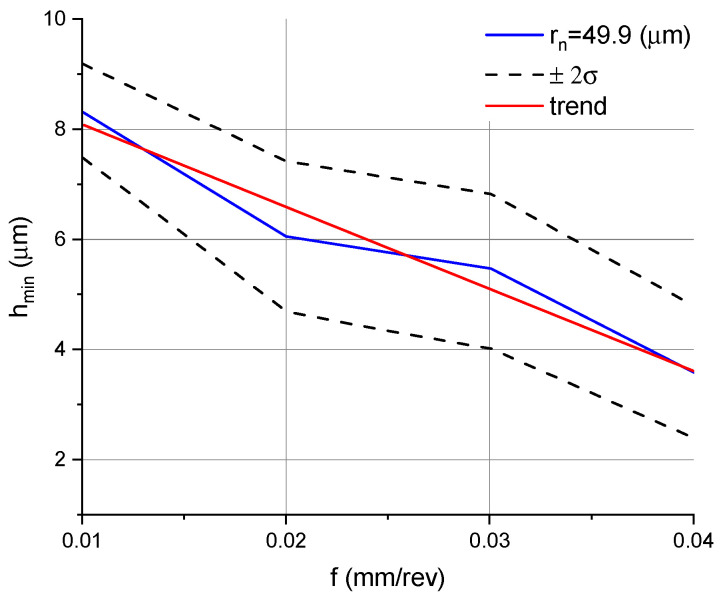
Minimum chip thickness vs. feed for the DCMT 11 T3 08–PF 4325 insert with a 49.9 µm edge radius.

**Figure 6:**
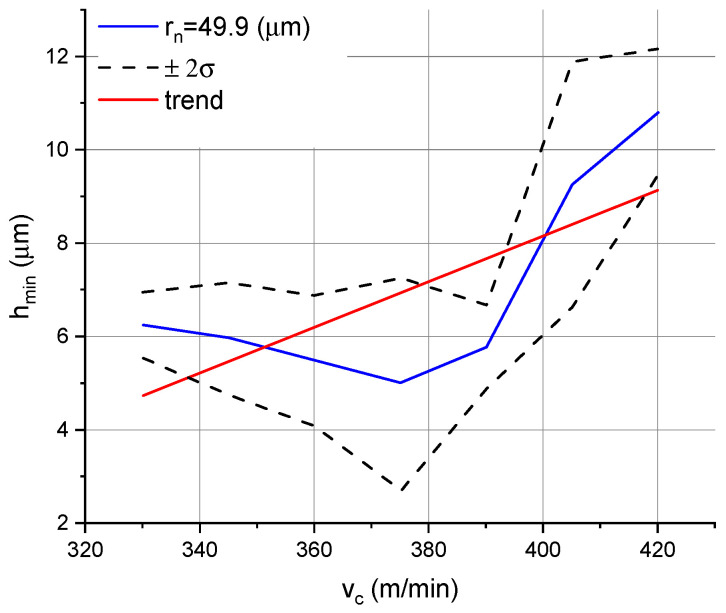
Minimum chip thickness vs. cutting speed for the DCMT 11 T3 08–PF 4325 insert with a 49.9 µm edge radius.

The data given in Table 2 were then used to illustrate how the parameter *h_min_* was dependent on the feed rate and cutting speed. The relationship between *h_min_* and the feed rate for the DCMT 11 T3 08–PF 4325 insert with a 49.9 µm edge radius is shown in Figure 5, while the relationship between *h_min_* and the cutting speed for the same insert type is given in Figure 6. When longitudinal turning was performed using a DCMT 11 T3 08–MF 1105 insert with an edge radius of 30 µm, the parameter *h_min_* was read only at feed rates lower than 0.02 mm/rev. Since no characteristic zones were identified at the feeds tested for the DCGT 11 T3 08–UM 1125 inserts with *r_n_* = 7.8 µm, this type of insert will not be discussed further in this article.

From the results provided in Figure 5, it is evident that *h_min_* decreases with decreasing feed rate. The parameter could not be read at feed rates greater than the edge radius. Low values for the feed rate, depth of cut, and edge radius resulted in ploughing and elastic and plastic deformation of the material that was removed. When the cutting parameters increased, the elastic and plastic deformation decreased and the material was removed as chips, with the chip thickness being equal to the depth of cut. A decrease in *h_min_* with increasing feed rate was due to an increase in the cross-sectional area of the cut material.

Figure 6 shows the effect of the cutting speed on the parameter *h_min_*. A decrease in *h_min_* was reported at speeds ranging from 330 to 375 m/min; with a faster cutting speeds, the parameter increased. This phenomenon may have been due to an increase in the edge radius as a result of insert wear. Faster cutting speeds caused faster tool wear. It is important to note that one insert was used to test each insert type across the whole range of cutting speeds. In Figure 6, the red straight line representing the average values of the parameter *h_min_* over the whole cutting speed range increases, which allows us to conclude that the minimum chip thickness increases with increasing cutting speed.

**Figure 7:**
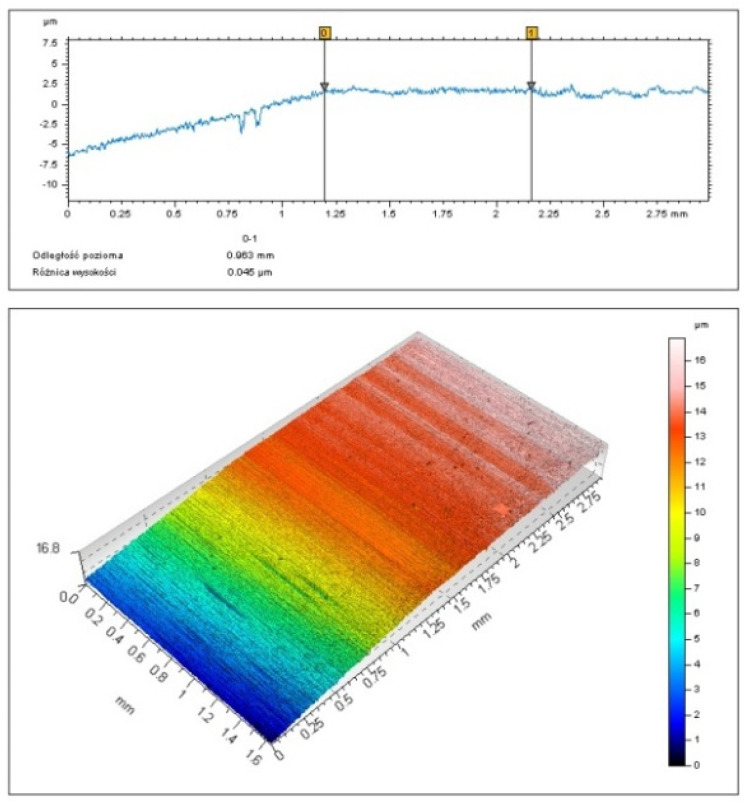
A 3D surface image and the corresponding 2D profile for a sample cut at *v_c_* = 360 m/min and *f =* 0.03 mm/rev using an insert with an edge radius of 49.9 µm.

**Figure 8:**
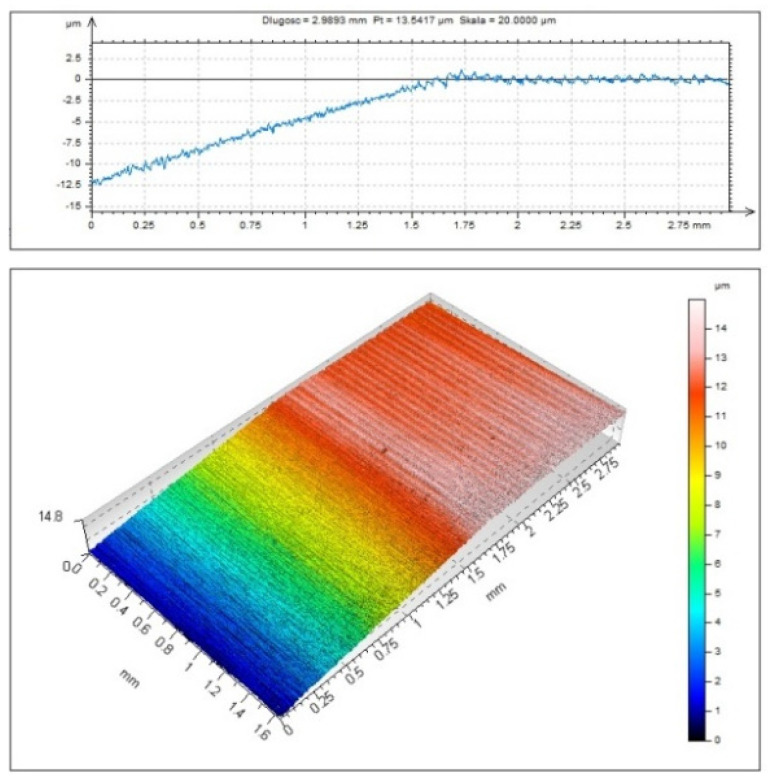
A 3D surface image and the corresponding 2D profile for a sample cut at *v_c_* = 360 m/min and *f* = 0.09 mm/rev using an insert with an edge radius of 49.9 µm.

The test results confirm that the minimum chip thickness is dependent on the edge radius. The edge radius changes due to the wear of the tool associated with machining. The method for determining the minimum chip thickness proposed in this article requires analyzing the primary surface profile. The analysis takes into account the tool wear, as it is assumed to contribute to an increase in *h_min_*. To ensure appropriate interpretation of the test results concerning the parameter *h_min_*, it was essential to analyze the machined surfaces using an optical profilometer to identify the zones of the interaction between the tool and the workpiece. The data obtained for an insert measuring 49.9 µm are illustrated in Figure 7 and Figure 8.

Figure 7 shows a 3D surface image and the corresponding 2D profile for a sample cut at a feed rate of 0.03 mm/rev using an insert with an edge radius of 49.9 µm. As can be seen, zone 1 represents the ground surface. The first interaction of the tool with the workpiece is clearly visible. The moment is marked as point 0 on the 2D profile. Then, up to point 1, no regular profile typical of turning with chip removal was observed. This suggests that the material undergoes elastic and plastic deformation and that ploughing occurs. The phenomena are also visible in the 3D image of the surface. The transition region is 0.96 mm in length, with the value being the same as that determined with a contact profilometer. The same analysis was carried out for the sample illustrated in Figure 4. The results of non-contact 3D measurements are shown in Figure 8.

Figure 4 and Figure 8 reveal that turning at a feed rate of 0.09 mm/rev using a tool with an edge radius of 49.9 µm does not cause any visible elastic and plastic deformation. This is due to the fact that the first tool–workpiece interaction was sufficient for chip formation. The 2D profile shows that once the tool interacts with the workpiece, the profile becomes regular, which is characteristic of turning with chip removal.

As the chip thickness is largely dependent on the feed rate during turning, the minimum chip thickness must be defined as the minimum feed rate at which a chip is formed. 

In most studies, *h_min_* is assumed to be less than or equal to 0.4 of the edge radius. In this article, the minimum chip thickness is determined as the point on the profile where the irregularity ends. When the cutting speed and feed increase, the elastic and plastic deformation of the layer removed from the workpiece decreases. For the DCMT 11T3-MF 1105 insert, *h_min_* was read at a maximum feed (0.02 mm/rev) and was 0.69 *r_n_*; for the DCMT 11 T3 08 insert, the value of *h_min_* read at a maximum feed (0.04 mm/rev) was 0.17 *r_n_*. High values of the parameter *h_min_* may have been due to the wear of the cutting edge (an increase in the edge radius), as wear is responsible for larger cutting edge and tool nose radii.

Comparing the values of the parameter *h_min_* obtained in the experiment with those calculated using the different models available in the literature is difficult because it requires the determination of many constants used in these formulae. Additionally, some of the models are dedicated to specific materials or tool–material systems.

## 4. Conclusions

The following conclusions were drawn from the experimental results:

1. The method employed to measure *h_min_* requires that a workpiece with a simple shape and a high-resolution instrument be used to measure surface profiles so that the characteristic tool–workpiece interaction zones can be defined easily and accurately;
2. The method overcomes the drawbacks of radial run-out during turning by supporting the workpiece on a mandrel;
3. A non-contact 3D optical profilometer can be sued as an alternative to the contact skidless profilometer used in the experiment;
4. The parameter *h_min_* is dependent on the cutting edge geometry, depth of cut, and feed rate. For the DCMT 11T3–MF 1105 insert, *h_min_* was read at a maximum feed (0.02 mm/rev) and was 0.69 *r_n_*; for the DCMT 11 T3 08 insert, the value of *h_min_* read at a maximum feed (0.04 mm/rev) was 0.17 *r_n_*;
5. The parameter *h_min_* was affected by the feed rate and cutting speed. For 0 < *f* < *r_n_*, an increase in the feed rate caused a decrease in the minimum chip thickness *h_min_^,^*; higher cutting speeds were responsible for higher values of *h_min_*;
6. The parameter *h_min_* can be used as an auxiliary parameter to determine the wear of the cutting edge. The minimum chip thickness increases with increasing edge radius;
7. The values of the parameter *h_min_* were determined for samples made of X37CrMoV5-1 steel. They are likely to be different for other tool and workpiece materials, as hardness, whether of the workpiece or the tool, is a crucial parameter in cutting.

## Figures and Tables

**Table 1:** Models used to calculate the minimum chip thickness.

No.	Model	Reference
1.	$h_{min}=r_{n}\left(1-\cos\theta \right)$	[12]
2.	$h_{min}=k\;r_{n}$	[13]
3.	$h_{min}\geq 0.5\;r_{n}\left[1-\frac{2\tau_{a}}{Y_{ch}}\right]$	[14]
4.	$h_{min}=r_{n}\left(1-\frac{Fy+\mu Fx}{\sqrt{(Fx^{2}+Fy^{2})\left(1+\mu^{2}\right)}}\right)$	[15]
5.	$h_{min}=r_{n}\left(1-\cos\left(\frac{\pi }{4}-\frac{\beta }{2}\right)\right)$	[16,17]
6.	$h_{min}=r_{n}\left[1-\cos\left(arc\;ctg\frac{A_{f}}{A_{c}}\right)\right]$	[18]

**Table 2:** Cutting conditions for the three insert types.

DCMT 11 T3 08–PF 4325, *r_n_* = 49.9 µm									
No.	*v_c_* [m/min]	*f* [mm/rev]	*n* [rev/min]	*h_min_* [µm]	No.	*v_c_* [m/min]	*f* (mm/rev)	*n* [rev/min]	*h_min_* [µm]
1.	360	0.01	2349	8.37 ± 0.87	7.	330	0.03	2167	6.24 ± 0.71
2.	360	0.02	2349	6.07 ± 1.39	8.	345	0.03	2265	5.95 ± 1.21
3.	360	0.03	2349	5.47 ± 1.39	9.	360	0.03	2364	5.74 ±1.19
4.	360	0.04	2349	3.61 ± 1.21	10.	375	0.03	2462	4.98 ± 2.27
5.	360	0.05	2349	----------	11.	390	0.03	2561	5.77 ± 0.89
6.	360	0.06	2349	----------	12.	405	0.03	2659	9.24 ± 2.63
					13.	420	0.03	2758	10.79 ± 1.35
DCMT 11 T3 08–MF 1105, *r_n_* = 30 µm					DCGT 11 T3 08–UM 1125, *r_n_* = 7.8 µm				
14.	360	0.01	2349	20.56 ± 5.37	20.	360	0.01	2349	---------
15.	360	0.02	2349	6.93 ± 3.81	21.	360	0.02	2349	---------
16.	360	0.03	2349	---------	22.	360	0.03	2349	---------
17.	360	0.04	2349	---------	23.	360	0.04	2349	---------
18.	360	0.05	2349	---------	24.	360	0.05	2349	---------
19.	360	0.06	2349	---------	25.	360	0.06	2349	---------

## Nomenclature

*v_c_*	[m/min] cutting speed
*f*	[mm/rev] feed rate
*a_p_*	[mm] depth of cut
*n*	[rev/min] spindle speed
*h_min_*	[μm] minimum chip thickness
*r_n_*	[μm] cutting edge radius
*r_ε_*	[mm] tool nose radius
*τ_a_*	[MPa] shear strength in the adhesive junction
*Y_ch_*	[MPa] yield stress due to strain hardening
*k*	[-] coefficient of friction
*μ*	[-] coefficient of friction
*F_x_*	[N] horizontal force
*F_y_*	[N] vertical force
*A_f_*	[-] directional coefficients
*A_c_*	[-] directional coefficients
*β*	[°] friction angle between the tool and the workpiece
*π*	[°] friction angle
*θ*	[°] stagnant angle
